# Adoptive cell therapy with tumor infiltrating lymphocytes and intermediate dose IL-2 for metastatic melanoma

**DOI:** 10.1186/2051-1426-2-S3-P1

**Published:** 2014-11-06

**Authors:** Rikke Andersen, Marco Donia, Troels Holz Borch, Eva Ellebæk Steensgaard, Trine Zeeberg Iversen, Per Kongsted, Mads Hald Andersen, Per thor Straten, Inge Marie Svane

**Affiliations:** 1 Center for Cancer Immune Therapy, Dept. of Hematology and Dept. of Oncology, Copenhagen University Hospital, Herlev, Denmark, Herlev, Denmark; 2 Center for Cancer Immunotherapy, Herlev, Denmark

## Background

Adoptive cell therapy (ACT) with tumor infiltrating lymphocytes (TILs) achieved impressive clinical results in several single institution Phase I/II clinical trials performed outside of Europe, and holds the promise to enter the mainstream of standard melanoma care in the near future. However, although transient, the toxicities associated with high-dose interleukin-2 (IL-2) classically administered together with TILs are severe and recent results have questioned its use. To further scrutinize IL-2 dosing, we have carried out a Phase II trial using TILs after classical lymphodepletion but followed by an attenuated regimen of IL-2.

## Materials and methods

A total of 25 patients with progressive metastatic melanoma, PS ≤1, age <70 and at least one resectable metastasis was included in this Phase II study (NCT00937625). TIL infusion was preceded by standard lymphodepleting chemotherapy but followed by an intravenous intermediate dose IL-2 decrescendo regimen.

## Result

The trial is fully recruited. Data indicate that the lower dose of IL-2 considerably decreased the toxicity of the treatment, and imaging evaluations of the first 22 patients evaluated showed two complete responses (29+, 16+ months) and seven partial responses (28+, 12, 20+, 12 and 11+, 8, 6+ months) with most responses still ongoing. Clinical responses were associated with high numbers of tumor reactive T cells infused. Importantly, in most responding patients we observed induction and durable persistence of anti-melanoma T cell responses in the peripheral blood. Updated results will be presented at the 2014 SITC conference.

## Conclusions

As the first European institution we show that TIL-based ACT is reliable, logistically feasible to administer and clinically effective in metastatic melanoma. Importantly, a high response rate including long-lasting complete responses can be induced after treatment with TILs followed by an attenuated regimen of IL-2, which considerably reduced the occurrence of severe side effects. Effective TIL treatment is associated with induction and long-term persistence in the blood of T cells producing in vitro anticancer responses.

